# Editorial: Emerging trends in moyamoya disease: diagnostic and therapeutic innovations

**DOI:** 10.3389/fneur.2026.1796066

**Published:** 2026-05-29

**Authors:** Shihao He, Hansen Chen, Rong Wang, Yuanli Zhao

**Affiliations:** 1 Department of Pathology, Johns Hopkins School of Medicine, Baltimore, MD, United States; 2 Department of Neurosurgery, Peking Union Medical College Hospital, Chinese Academy of Medical Sciences and Peking Union Medical College, Beijing, China; 3 Department of Neurosurgery, School of Medicine, Stanford University, Stanford, CA, United States; 4 Department of Neurosurgery, Beijing Tiantan Hospital, Capital Medical University, Beijing, China

**Keywords:** epigenetic regulation, imaging biomarkers, moyamoya disease, neuropsychological outcomes, revascularization, serum biomarkers

This Research Topic explores recent advances in the diagnosis, mechanistic understanding, and treatment of moyamoya disease. Moyamoya disease (MMD) is a rare, progressive cerebrovascular disorder characterized by chronic stenosis or occlusion of the terminal internal carotid arteries and the development of fragile collateral networks. In moyamoya disease, angiographic results and surgical patency have long served as primary outcome measures, but they no longer capture the full spectrum of perioperative risk and post-operative neurological recovery that determines patient prognosis.

This Research Topic aims to explore the latest trends in moyamoya disease diagnosis, mechanistic understanding, and treatment innovations. This field is rapidly evolving, moving beyond traditional angiography descriptions and surgical paradigms. Rather than focusing on a single dimension of disease management, this Research Topic brings together eight articles integrating novel imaging strategies, serum biomarkers, mechanistic studies, and outcome-focused clinical research, illustrating how mechanistic discovery and clinical innovation are converging to reshape research priorities and therapeutic decision-making in moyamoya disease.

## Emerging roles of epigenetic regulation in moyamoya disease pathogenesis

Epigenetic mechanisms have received relatively limited attention in studies of moyamoya disease. Although RNF213 is widely recognized as a major susceptibility gene, its role as an E3 ubiquitin ligase raises the possibility that alterations in ubiquitination related processes may contribute to disease development in ways that are not explained by genetic variation alone. In this context, Niu et al. conducted one of the first systematic investigations focusing on ubiquitination-associated genes in MMD by integrating transcriptomic datasets, machine learning algorithms, and experimental validation. Their study identified ANAPC11, UCHL1, and USP41 as core ubiquitination-related genes, demonstrated reduced serum UCHL1 levels in MMD patients, and showed that UCHL1 deficiency promotes vascular smooth muscle cell migration. Furthermore, immune infiltration analyses revealed significant associations between these genes and interferon-related immune pathways, highlighting a potential link between ubiquitination, immune dysregulation, and vascular remodeling in moyamoya disease.

## Surgical innovation and translational refinement of revascularization techniques

Although cerebral blood flow reconstruction is widely used in moyamoya disease, there is no single approach that fits all patients. Decisions regarding direct, indirect, or combined bypass, as well as the use of adjunct procedures, often depend on anatomical and clinical factors that vary across individuals. Chen, Huang et al. provide a structured synthesis of cerebral blood flow reconstruction technologies and surgical strategies, emphasizing indications, technique selection, and expected therapeutic effects across different approaches. This article is particularly valuable in a Research Topic themed around “diagnostic and therapeutic innovations,” because it reframes bypass not as a single procedure but as a configurable set of techniques that must be matched to anatomy, hemodynamics, and clinical presentation (Chen, Huang et al.).

This deficiency naturally points to the need for future pragmatic clinical trials, the establishment of multicenter registries, and the standardization of reporting hemodynamic endpoints. Post-operative transient neurological deficits remain among the most clinically challenging and relevant and clinically relevant complications after revascularization. In this Topic, Liu et al. focus on post-operative motor aphasia following combined revascularization and introduce temporal muscle thickness (TMT) measured on preoperative MRI as a predictor of both the occurrence and duration of aphasia. The key conceptual contribution is the proposed biomechanical mechanism: temporalis swelling and local compression after surgery may contribute to transient deficits, meaning that not all post-operative symptoms should be interpreted purely through the lens of ischemia/hypoperfusion (Liu et al.). TMT is inexpensive, non-invasive, and implementable. In practice, this measure can inform decisions made before and after surgery, including operative technique, post-operative surveillance, and patient counseling. Rather than changing existing paradigms, it provides additional information that may help anticipate post-operative neurological symptoms in selected patients.

Shi et al. conducted a prospective cohort study comparing side to side and end to side STA to MCA bypass in 50 adult moyamoya hemispheres, with an emphasis on intraoperative hemodynamics measured by microvascular Doppler ultrasonography and peri discharge CT perfusion metrics. The two groups were similar in baseline characteristics, post-operative complication rates, bypass patency, Matsushima grades, and 6 month functional outcomes, and post-operative CT perfusion showed no clear between group differences in infarct core, hypoperfusion, or penumbra volumes. The main distinctions were technical and physiological. Side to side bypass required a longer bypass time and a larger anastomosis relative to the recipient artery, and Doppler measurements suggested higher flow velocity in the recipient artery segment entering the Sylvian fissure, along with higher fold changes in donor vessel velocity. The authors further described two intraoperative flow patterns observed in side to side bypass, termed flow shunting and flow augmentation, and proposed that anastomosis size and pressure gradients may contribute to these patterns. Overall, this study does not establish superiority of one technique over the other in short term clinical or perfusion outcomes, but it provides a structured way to quantify intraoperative flow behavior and links it to practical technical variables that surgeons can observe and potentially modify when selecting or performing direct bypass in adult moyamoya disease.

## Revisiting asymptomatic moyamoya: natural history, risk stratification, and therapeutic uncertainty

Management of asymptomatic moyamoya disease remains one of the most challenging and controversial areas in clinical practice, largely due to the limited understanding of its natural history and the uncertain benefit of preventive surgical intervention. In this Research Topic, Chen, Zhou et al. conducted a comprehensive systematic review and meta analysis to delineate the clinical and radiological course of asymptomatic and hemodynamically stable moyamoya disease. By synthesizing data from seven adult asymptomatic cohorts involving 393 patients and 649 hemispheres, the authors demonstrated that so called asymptomatic disease is not a benign condition. During a follow up period exceeding 2 years, conservatively managed patients exhibited a measurable risk of clinical progression, including ischemic and hemorrhagic stroke, as well as a substantial rate of radiological progression. Importantly, although revascularization was associated with numerically lower rates of stroke and radiological progression, pooled analyses did not demonstrate a statistically significant protective effect within the available follow up window. These findings underscore that symptom status alone is insufficient for risk stratification in moyamoya disease and highlight the urgent need for more refined predictors of disease progression. Rather than advocating routine preventive surgery, this work emphasizes the importance of long term surveillance and the development of reliable imaging and biological markers to identify asymptomatic patients who are at higher risk of future clinical deterioration and may benefit from earlier intervention (Chen, Zhou et al.). Future studies addressing revascularization in asymptomatic disease would also benefit from systematic evaluation of antithrombotic strategies, perioperative hemorrhagic risk, and long-term stroke prevention, areas in which current evidence remains limited and heterogeneous.

## Serum biomarkers and perioperative ischemic risk

Wu et al. examined the relationship between perioperative low density lipoprotein levels and post-operative cerebral infarction in adult patients with moyamoya disease. Instead of using a single baseline measurement, the authors analyzed LDL values obtained at multiple perioperative time points and derived several summary indicators reflecting post-operative change. Using these variables, they constructed a predictive model for post-operative infarction and showed that LDL related measures remained independently associated with risk after adjustment for clinical factors. Perioperative LDL fluctuation, in particular, may serve as a surrogate marker of metabolic and inflammatory instability during the surgical period, which could increase vulnerability to ischemic injury in a hemodynamically fragile cerebrovascular system. Although these mechanistic links still need to be confirmed, this study supports the concept that a dynamic perioperative biomarker profile (rather than just static baseline values) can improve short-term risk stratification after revascularization procedures. This approach highlights that perioperative metabolic fluctuations, rather than baseline lipid status alone, may be relevant to short term ischemic outcomes after revascularization. From a clinical perspective, the study demonstrates how routinely collected laboratory data can be incorporated into risk models to support perioperative assessment, although the biological mechanisms linking LDL dynamics to infarction risk in moyamoya disease remain to be clarified (Wu et al.). Future studies could combine lipid profiles with markers of endothelial activation, platelet function, and perfusion imaging endpoints.

Complementing LDL-focused metabolic risk, Sun et al. evaluate serum alkaline phosphatase (ALP) levels in MMD and link higher ALP to poorer early post-operative outcomes after revascularization. The clinical appeal of ALP is similar to LDL: it is widely available, low-cost, and routinely measured. If validated externally, it could become part of a practical perioperative risk panel. ALP has been implicated in vascular calcification and endothelial function in broader cardiovascular literature, which may align with the Topic's emphasis on connecting molecular/biochemical signals to clinical practice (Sun et al.). This study suggested that MMD is a cerebrovascular disorder, systemic vascular and metabolic milieus may influence surgical vulnerability and recovery. The next step is prospective validation and incorporation into multi-marker models that include imaging and clinical features. Together, these two studies highlight the potential value of readily accessible serum biomarkers in capturing systemic vascular and metabolic milieus that may modulate perioperative vulnerability and recovery in moyamoya disease.

## Neuropsychological outcomes and post-operative recovery in adult moyamoya disease

Liang et al. examined changes in psychological status, cognitive function, and health related quality of life in a cohort of adult, surgically treated Chinese patients with symptomatic moyamoya disease undergoing revascularization surgery. Using validated instruments including the Hospital Anxiety and Depression Scale, Montreal Cognitive Assessment, and the 36-Item Short Form Health Survey, the authors assessed patients both before surgery and during post-operative follow up. Their analysis showed that, at the group level, revascularization was associated with reductions in anxiety and depressive symptoms, as well as improvements in several domains of quality of life, particularly among patients who exhibited preoperative psychological or cognitive impairment. Post-operative changes in psychological and quality of life measures varied substantially among patients. While improvements were observed at the group level, individual post-operative scores showed considerable dispersion, and some domains changed only modestly during follow up. These findings indicate that neuropsychological recovery after revascularization in moyamoya disease is not uniform and cannot be assumed for all patients. By documenting these measures alongside standard clinical outcomes, the study adds granularity to post-operative evaluation and supports the inclusion of repeated cognitive and psychological assessments in longitudinal moyamoya cohorts.

## Conclusion

This research Research Topic focuses on how diagnostic innovations, computational discoveries, and surgical advancements are collectively driving transformative changes in the diagnosis and treatment of moyamoya disease. These eight articles cover imaging biomarkers, serum and metabolic predictors, evidence synthesis for challenging clinical populations, the evolution of revascularization techniques, and patient-centered outcome assessments, all contributing to the field's progression toward a more precise, comprehensive, and translational paradigm. We hope this Research Topic of articles will inspire further interdisciplinary collaboration and accelerate the development of diagnostic and therapeutic approaches, ultimately improving the long-term prognosis for patients with moyamoya disease.

